# UHPLC-UV/PDA Method Validation for Simultaneous Quantification of Luteolin and Apigenin Derivatives from *Elaeis guineensis* Leaf Extracts: An Application for Antioxidant Herbal Preparation

**DOI:** 10.3390/molecules26041084

**Published:** 2021-02-19

**Authors:** Mohamad Shazeli Che Zain, Muhamad Faris Osman, Soo Yee Lee, Khozirah Shaari

**Affiliations:** Natural Medicines and Products Research Laboratory (NaturMeds), Institute of Bioscience, Universiti Putra Malaysia, Serdang 43400, Malaysia; shazelizain@gmail.com (M.S.C.Z.); farisosman@iium.edu.my (M.F.O.); daphne.leesooyee@gmail.com (S.Y.L.)

**Keywords:** oil palm leaves, luteolin and apigenin derivatives, UHPLC-UV/PDA method validation, simultaneous quantification, antioxidant activity, partial least square analysis

## Abstract

Luteolin and apigenin derivatives present in oil palm (*Elaeis guineensis*) leaves (OPL) are reported to possess excellent antioxidant properties relating to numerous health benefits. To meet the global demand for flavonoids, OPL, which is plentifully generated as an agricultural by-product from oil palm plantations, can be further exploited as a new source of natural antioxidant compounds. However, to produce a standardized herbal preparation, validation of the quantification method for these compounds is required. Therefore, in this investigation, we developed and validated an improved and rapid analytical method, ultra-high-performance liquid chromatography equipped with ultraviolet/photodiode array (UHPLC-UV/PDA) for the quantification of 12 luteolin and apigenin derivatives, particularly focusing on flavonoid isomeric pairs: orientin/isoorientin and vitexin/isovitexin, present in various OPL extracts. Several validation parameters were assessed, resulting in the UHPLC-UV/PDA technique offering good specificity, linearity, accuracy, precision, and robustness, where the values were within acceptable limits. Subsequently, the validated method was employed to quantify luteolin and apigenin derivatives from OPL subjected to different drying treatments and extraction with various solvent systems, giving total luteolin (TLC) and apigenin content (TAC) in the range of 2.04–56.30 and 1.84–160.38 µg/mg extract, respectively. Additionally, partial least square (PLS) analysis disclosed the combination of freeze dry-aqueous methanol yielded OPL extracts with high TLC and TAC, which are strongly correlated with antioxidant activity. Therefore, we provide the first validation report of the UHPLC-UV/PDA method for quantification of luteolin and apigenin derivatives present in various OPL extracts, suggesting that this approach could be employed in standardized herbal preparations by adopting orientin, isoorientin, vitexin, and isovitexin as chemical markers.

## 1. Introduction

The oil palm (*Elaeis guineensis* Jacq.) tree is a primary industrial crop in many countries, including Malaysia and Indonesia, due to the high potential of its fruits to produce edible oils. The huge oil plant plantations generate an abundance of by-products, including oil palm leaves (OPL). Interestingly, OPL is a natural source of flavonoids that possess functional properties for disease prevention [1,2,3]. Prior research on the metabolite profile of OPL highlighted the existence of flavonoid *C*-glycosides, i.e., catechin, luteolin, and apigenin derivatives that have an array of therapeutic properties, including anti-inflammatory, antioxidant, and wound healing properties [4,5,6,7,8,9]. Recently, OPL has been developed as an herbal tea product in Malaysia for daily consumption (http://fyllo.com.my/). To produce a standardized OPL herbal preparation, nutraceutical and pharmaceutical industries demand a validated analytical method for rapidly detecting and quantifying these compounds.

The ultra-high-performance liquid chromatography coupled with ultraviolet/photodiode array (UHPLC-UV/PDA) technique is a powerful and systematic tool that is widely employed in separating and quantifying flavonoids present in complex mixtures [10,11]. To consider the method as validated for quantification purpose, the International Conference on Harmonization (ICH) provided guidelines comprising several parameters for assessment, including sensitivity, linearity, limits of detection and limits of quantification (LOD and LOQ, respectively), accuracy, precision, and robustness [12]. The application of the validated UHPLC-UV/PDA method for simultaneous flavonoid quantification has been established for various plant extracts, including *Dracocephalum heterophyllum* Benth. [13], *Myrcia uniflora* [14], *Dimorphandra gardneriana* [15], *Trigonella stellate* [11], and many others [16,17,18,19,20,21]. The identification of flavonoid *C*-glycosides in OPL via UHPLC-UV/PDA-MS/MS analysis has been described in our previous works [4,5,6]. Tahir et al. (2012) established a method with the primary aim of comprehensively profiling luteolin and apigenin derivatives present in OPL. The initial method for analysis of the compounds was considered too lengthy and impractical for industrial application [6]. Hence, an improved and rapid method for simultaneous quantification of luteolin and apigenin derivatives in various OPL extracts is required. However, the development of a rapid UHPLC-UV/PDA quantification method for OPL samples became a challenge due to the presence of isomeric pairs of the target flavonoids viz. orientin/isoorientin and vitexin/isovitexin, which have similar molecular weights and eluted at the same retention times. Recently, we developed an improved method in which the analysis time was reduced significantly [4,5]. Herein, we report the validation of the rapid detection and quantification method.

Furthermore, to produce a standardized OPL extract containing the optimal level of antioxidant compounds, the processing conditions, including a drying treatment and the solvent system for extraction, should be properly selected. Studies showed these processing conditions greatly affected the preservation and recovery of antioxidant compounds from various plant materials, such as *Phyllanthus niruri* [22], *Brassica oleracea* L. [23], *Phoenix dactylifera* [24], and *Neptunia oleracea* [25]. As for OPL, to the best of our knowledge, the effects of different drying methods and solvents on the recovery of the antioxidant compounds such as luteolin and apigenin derivatives have yet to be explored.

**Figure 1:**
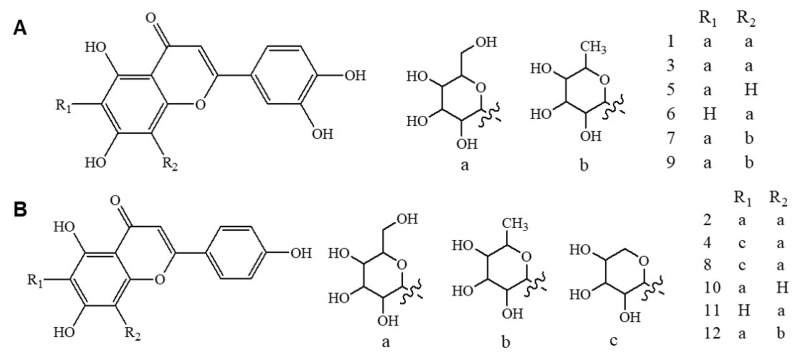
Chemical structures of luteolin derivatives (**A**) and apigenin derivatives in oil palm leaves (OPL) extract (**B**). Compound assignment: **1**, luteolin-6,8-di-*C*-hexose (Isomer 1); **3**, luteolin-6,8-di-*C*-hexose (Isomer 2); **5**, isoorientin; **6**, orientin; **7**, luteolin-6-*C*-hexose-8-*C*-deoxyhexose (Isomer 1); **9**, luteolin-6-*C*-hexose-8-*C*-deoxyhexose (Isomer 2); **2**, apigenin-6,8-di-*C*-hexose; **4**, apigenin-6-*C*-pentose-8-*C*-hexose (Isomer 1); **8**, apigenin-6-*C*-pentose-8-*C*-hexose (Isomer 2); **10**, vitexin; **11**, isovitexin; and **12**, apigenin-6-*C*-hexose-8-*C*-deoxyhexose.

Therefore, the present study was primarily designed to develop and validate the UHPLC-UV/PDA method for simultaneous quantification of identified luteolin and apigenin derivatives present in various OPL extracts, particularly focusing on four target compounds: isoorientin, orientin, vitexin, and isovitexin. To validate the proposed analytical method, parameters including sensitivity, linearity, detection, and quantification limits (LOD and LOQ), accuracy, precision, and robustness were assessed. Upon validation, the method was applied to quantify 12 luteolin and apigenin derivatives present in various OPL extracts prepared from differently dried samples and using different solvent systems (Figure 1). In the final part of the study, the prepared OPL extracts containing the optimal level of luteolin and apigenin derivatives were correlated with antioxidant activity by employing partial least square (PLS) analysis. The findings highlighted the potential use of luteolin and apigenin derivatives, particularly orientin, isoorientin, vitexin, and isovitexin, as chemical markers for quality control of standardized OPL herbal extract preparation.

## 2. Materials and Methods

### 2.1. Chemicals and Reagents

Antioxidant assay reagents quercetin, ammonium formate, and formic acid were bought from Sigma-Aldrich (St. Louis, MO, USA). Conventional organic solvents were supplied by R & M Chemicals (Essex, UK). The MilliQ system was used to produce deionized water. Analytical-grade acetonitrile and water were acquired from Merck (Darmstadt, Germany). The standards of vitexin, isovitexin, orientin, and isoorientin with purity >98.0% were provided by ChemFaces (Wuhan, China).

### 2.2. Preparation of Various OPL Extracts

Oil palm leaves were harvested from University Agricultural Park, Universiti Putra Malaysia (UPM), Malaysia. For uniformity in leaf sampling, the 16th, 17th, and 18th fronds were harvested. The leaflets were separately detached from the petiole and the mid-ribs were removed and cut into small pieces. The OPL was oven-dried (O) at 35 °C, freeze-dried (F) at 0.064 mbar and −50 °C, and shade-dried (S) at ambient temperature. The dried samples were pulverized and sieved to obtain uniform-sized OPL powders. Subsequently, the powders were extracted using solvent systems with varying polarities viz. aqueous methanol (4:1 methanol–water), absolute methanol, ethyl acetate–methanol (1:1), ethyl acetate, and hexane. Ultrasound-assisted extraction was employed for maximal liberation of luteolin and apigenin derivatives at optimal conditions [4]. The OPL extracts were recovered by vacuum evaporation.

### 2.3. Development of UHPLC-UV/PDA Method

The analysis was executed on the Dionex Ultimate 3000 UHPLC system fitted with a PDA-3000 photodiode array detector. To separate the compounds in OPL extract, an Acquity UPLC^®^ BEH C_18_ column with 1.7 µm particle size, 100 mm length, and 2.1 mm internal diameter. The elution of compounds was assisted with two mobile phase systems comprising solvent A (water, 0.1% formic acid, and 0.063% ammonium formate) and solvent B (acetonitrile and 0.1% formic acid). The programmed gradient progressed using the subsequent order of solvent B (%): 10% for 0–3 min, 10–11% for 3–5 min, 11–11.3% for 5–7 min, 11.3–11.4% for 7–12 min, 11.4–11.8% for 12–12.2 min, 11.8–12% for 12.2–18 min, 12–10% for 18–19 min, and 10% for 19–20 min. The flow rate was set to 0.40 mL/min. The PDA wavelength (λ) was set to the range of 200–400 nm and the UV channels were set to 270 and 340 nm. The UHPLC-UV/PDA results were analyzed using Thermo Scientific Fisher Qual Browser Xcalibur^®^ software. The complexity of OPL crude extract made the baseline separation difficult. Hence, to obtain better peak resolution, particularly for the flavonoid *C*-glycosides, acid hydrolysis was performed on the crude extract according to our recent published method [5]. The hydrolyzed OPL extract was recovered by vacuum evaporation.

### 2.4. UHPLC-MS/MS for Identification of Luteolin and Apigenin Derivatives

The UHPLC conditions were similar, as mentioned earlier, while the MS analysis was operated on a Thermo Scientific™ Q Exactive™ Hybrid Quadrupole-Orbitrap mass spectrometer with a 200 µL flow rate for electrospray ionization (ESI). The eluted compound was controlled under negative mode scanned from *m*/*z* 67.9 to 1000. ESI was operated using a spray voltage of 4.2 kV. The capillary temperature was maintained at 320 °C while the auxiliary gas heater temperature was tuned to 0 °C. The UHPLC-MS/MS results were analyzed using Thermo Scientific Fisher Qual Browser Xcalibur^®^ software.

### 2.5. Identification of Luteolin and Apigenin Derivatives

In our previous studies, flavonoid *C*-glycosides in OPL were comprehensively identified based on UHPLC-MS/MS and UHPLC-UV/PDA analysis [4,5,6]. The peak assignment was done by comparing the molecular formula produced by isotope pattern, retention times (t_R_), mass-to-charge ratio (*m*/*z*), their fragmentation pattern via collision induced dissociation (CID), relative abundance of ions, and UV/vis absorption of some available standards (orientin, isoorientin, vitexin, and isovitexin). The results indicated that similar apigenin and luteolin derivatives were detected comprising isoorientin, luteolin-6,8-di-*C*-hexose, luteolin-6-*C*-hexose-8-*C*-deoxyhexose, apigenin-6-*C*-hexose-8-*C*-deoxyhexose, vitexin, apigenin-6,8-di-*C*-hexose, luteolin-6-*C*-hexose-8-*C*-deoxyhexose, apigenin-6-*C*-pentose-8-*C*-hexose, orientin, and isovitexin. The details of the compound identification are presented as Supplementary Material (Figure S1 and Table S1).

### 2.6. Validation of UHPLC-UV/PDA Method

#### 2.6.1. Specificity

The capability of a technique to differentiate between compounds of interest and other elements such as contaminants, impurities, and adducts in the tested sample describes the sensitivity, also known as the specificity, of the analytical method. In this study, the sensitivity of the UHPLC technique was demonstrated by injecting a procedural blank, commercial standards (isoorientin, orientin, vitexin, and isovitexin), and sample solution (OPL crude and hydrolyzed extracts). At the same retention time, the overlapping of peaks between the commercial standard and the peak that appeared in the tested samples was expected to have similar compounds. Additionally, the purity of the compounds was verified using PDA (λ = 200–400 nm) and isotope patterns (MS/MS). The goal **for** this parameter was to ensure there was no interference by other constituents at the peaks of interest.

#### 2.6.2. Linearity

The calibration line plotted by different concentrations of the commercial standard of a particular compound generates a correlation coefficient (R^2^) that can be used as an indicator for the linearity parameter of an analytical method. In this study, the calibration curves of isoorientin, isovitexin, orientin, and vitexin were generated by injecting six different concentration levels of standards separately. With 1000 µg/mL stock solution, the final concentrations of isoorientin and isovitexin were 500, 250, 125, 63, 31, and 16 µg/mL, whereas those of orientin were 800, 500, 250, 125, 63, and 31 µg/mL. With 1500 µg/mL stock solution, the final concentrations of vitexin were 1500, 750, 375, 188, 94, and 47 µg/mL. The triplicate values of each concentration were averaged and used to plot the calibration curves.

#### 2.6.3. Limit of Detection (LOD) and Limit of Quantification (LOQ)

Every analytical method provides LOD and LOQ information to describe the lowest concentrations that can be used to detect and quantify the compounds of interest present in tested samples. Referring to plotted calibration curves, the ratios of signal to noise set for LOD and LOQ were 3.3:1 and 10:1, respectively. These values were calculated by using the following formula:

LOD = (SD × 3.3)/IC(1)

LOQ = (SD × 10)/IC(2)

where SD is the standard deviation of the response and IC is the slope of the calibration curve.

#### 2.6.4. Accuracy

The accuracy of an analytical approach could be evaluated by performing recovery experiments. It was performed by adding known concentrations of flavonoid standards. Standard concentrations were prepared at three levels (high, medium, and low); isoorientin (500, 250, 125 mg/mL), orientin (500, 250, 125 mg/mL), isovitexin (500, 250, 125 mg/mL), and vitexin (750, 375, 188 mg/mL) were spiked into the blank sample. The recovery (%) of each sample was determined as follows:

Recovery (%) = (A_F_ − A_O_)/A_S_ × 100 (3)

where A_F_, A_O_, and A_S_ are the amount found, original amount, and amount added, respectively.

#### 2.6.5. Repeatability and Intermediate Precision

The repeatability (intra-day) and intermediate precision (inter-day) of the analytical method were assessed by repetitive injections. First, the intra-day precision was determined by performing three-time injections at three levels of concentrations (high, medium, and low) of flavonoids on the same day: isoorientin (500, 250, 125 mg/mL), orientin (800, 500, 250 mg/mL), isovitexin (500, 250, 125 mg/mL), and vitexin (1500, 750, 375 mg/mL). Intra-day precision was stated as the relative standard deviation (RSD) of the flavonoid concentrations.

Meanwhile, inter-day precision was validated by injecting three replicates of each concentration level of flavonoids for three consecutive days: isoorientin (500, 250, 125 mg/mL), orientin (800, 500, 250 mg/mL), isovitexin (500, 250, 125 mg/mL), and vitexin (1500, 750, 375 mg/mL). Inter-day precision was expressed as the RSD of the flavonoid concentrations. The RSD (%) was calculated as follows:

RSD (%) = (SD_F_ × 100)/AC_F_ × 100 (4)

where SD_F_ and AC_F_ are the flavonoid standard deviation and flavonoid average content, respectively.

#### 2.6.6. Robustness

The robustness of the analytical technique was investigated by conducting minor changes in the method conditions. The robustness of the UHPLC method was assessed by comparing the values obtained with different column temperatures (25 and 26 °C), wavelength detectors (340 and 342 nm), and on different days (Day 1 and Day 2). The significance of the changes was evaluated by performing *t*-test analysis, where *p* > 0.05 indicated non-significant difference of the set conditions.

### 2.7. Quantification of Luteolin and Apigenin Derivatives

The luteolin and apigenin derivatives detected in the OPL extracts derived from drying treatments and extraction solvents were quantified using the validated UHPLC-UV/PDA method. The contents of these flavonoids were analyzed using UV absorption data (area, mAU*min) recorded at the wavelength of 340 nm. Orientin, vitexin, isoorientin, and isovitexin were quantified absolutely, based on their respective calibration curves generated earlier, using commercial standards of the compounds. However, the amount of other luteolin and apigenin derivatives was relatively quantified. Luteolin derivatives were quantified relatively as orientin equivalents (µg/mg) using calibration curve for orientin, whereas apigenin derivatives were quantified relatively as vitexin equivalents (µg/mg) using a calibration curve for vitexin. Summing up the total amount of luteolin derivatives and apigenin derivatives yielded total luteolin content (TLC, µg/mg) and total apigenin content (TAC, µg/mg), respectively.

### 2.8. Determination of Total Phenolic Content

A total phenolic content (TPC) assay was performed using FC reagent and executed in a 96-well plate as explained earlier [25], with slight adjustments. Briefly, 20 µL of 0.1 mg/mL sample and 100 µL FC reagent were transferred into each well and incubated for 5 min. Subsequently, 80 µL of 7.5% sodium carbonate solution was added and the absorbance of the mixture was read at 750 nm using a micro-titer plate reader. Each sample was analyzed in triplicates. The TPC value was stated in milligrams of gallic acid equivalents per gram of extract (mg GAE/g extract).

### 2.9. Determination of Total Flavonoid Content

A total flavonoid content (TFC) assay was conducted using an aluminum chloride complex forming assay as reported formerly [4]. Briefly, in a 2 mL microcentrifuge tube, the mixture consisting of 125 µL of 0.1 mg/mL sample, 375 µL 95% ethanol, 25 µL 10% aluminum chloride solution, 25 µL 1M sodium acetate solution, and 700 µL distilled water was mixed homogenously and subjected to 40 min incubation for reaction to take place. A total of 200 µL of each mixture was transferred into each well of a 96-well plate prior to absorbance measurement at 415 nm using a micro-titer plate reader. Each sample was analyzed in triplicates. The TFC values were stated in milligrams of quercetin equivalents per gram of extract (mg QCE g^−1^ extract).

### 2.10. Determination of 2,2-Diphenyl-1-picrylhydrazyl (DPPH) Free Radical-Scavenging Activity

The DPPH-scavenging assay was executed as described previously [4]. Briefly, within a 96-well plate, 100 µg/mL of sample were serially diluted, with a final volume of 50 µL. After adding 100 µL of 0.059 mg/mL DPPH reagent solution into each well, the mixture was dark incubated for 30 min. The absorbance measurement was performed on a micro-titer plate reader at 515 nm. The DPPH-scavenging activity of commercial standards; gallic acid and quercetin were also tested and treated as positive controls. The scavenging activity (SA) was determined as:

SA % = (A_o_ − A_s_)/A_o_ × 100% (5)

where A_o_ and A_s_ are the reagent blank and sample absorbance values, respectively. Each sample was evaluated in triplicates. The values were stated as IC_50_ in microgram per milliliter (µg/mL).

### 2.11. Determination of Nitric Oxide (NO) Free Radical-Scavenging Activity

An NO-scavenging assay was performed as described previously [4]. Briefly, in a 96-well plate, 1000 µg/mL aliquots of the test samples were prepared and serially diluted to a final volume of 60 µL. After adding 60 µL of sodium nitroprusside solution into each well, the mixture was incubated for 150 min. The absorbance measurement was performed on a micro-titer plate reader at 550 nm after adding 60 µL Griess reagent solution. In the present investigation, Griess reagent was prepared by mixing 0.1 g sulphanilamide, 0.01 g *N*-(1-Naphthyl) ethylenediamine dihydrochloride, and 10 mL 2.5% phosphoric acid. The NO-scavenging activity of commercial standards; gallic acid and quercetin were also tested and treated as positive controls. The SA was determined according to Equation (5). Each sample was evaluated in triplicates. The values were stated as IC_50_ in microgram per milliliter (µg/mL).

### 2.12. Statistical Analysis

For the quantitative analysis of luteolin and apigenin derivatives, polyphenolic contents, and antioxidant activity, Minitab and GraphPad statistical software were applied. The values were shown as a mean ± standard deviation. To determine the significant difference of the values obtained, one-way analysis of variance (ANOVA) followed by Tukey’s test was performed, where *p* < 0.05 was set as the significant level. In addition, the correlation between luteolin and apigenin derivatives and antioxidant activity were executed by using a partial least square analysis (PLS) model. To perform the analysis, the IC_50_ values of antioxidant activity were transformed into 1/IC_50_ and the analysis was performed using UV scaling in SIMCA software.

## 3. Results and Discussion

### 3.1. Validation of Developed UHPLC-UV/PDA Method

#### 3.1.1. Specificity

**Figure 2:**
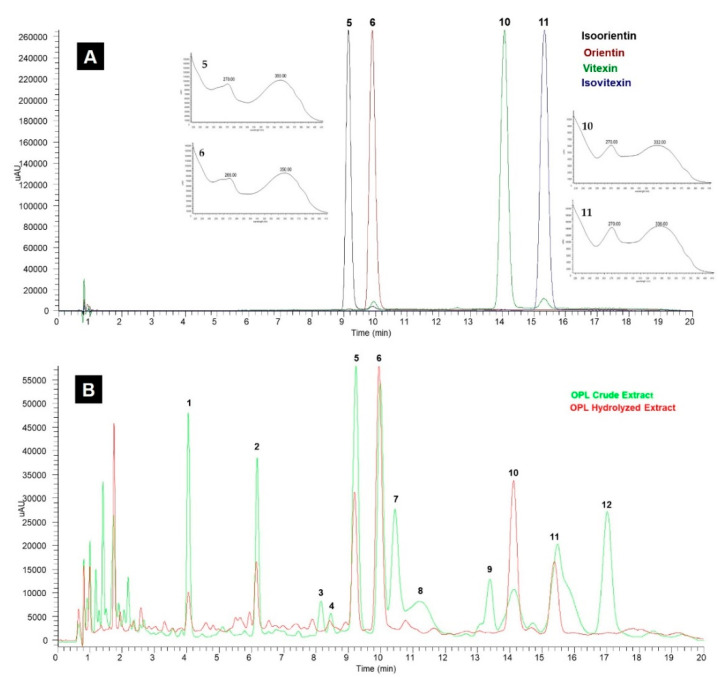
(**A**) Ultra-high-performance liquid chromatography-ultraviolet/photo-diode array (UHPLC-UV/PDA) chromatogram of commercial standards recorded at 340 nm: **5**, isoorientin; **6**, orientin; **10**, vitexin; and **11**, isovitexin. (**B**) Typical chromatogram of OPL crude and hydrolyzed extracts at 340 nm. Peak identified: **1**, luteolin-6,8-di-*C*-hexose (Isomer 1); **2**, apigenin-6,8-di-*C*-hexose; **3**, luteolin-6,8-di-*C*-hexose (Isomer 2); **4**, apigenin-6-*C*-pentose-8-*C*-hexose (Isomer 1); **5**, isoorientin; **6**, orientin; **7**, luteolin-6-*C*-hexose-8-*C*-deoxyhexose (Isomer 1); **8**, apigenin-6-*C*-pentose-8-*C*-hexose (Isomer 2); **9**, luteolin-6-*C*-hexose-8-*C*-deoxyhexose (Isomer 2); **10**, vitexin; **11**, isovitexin; and **12**, apigenin-6-*C*-hexose-8-*C*-deoxyhexose.

The sensitivity of the method was evaluated by comparing the peaks of commercial standards of isoorientin, orientin, vitexin, and isovitexin with the matching peaks in the OPL extracts. Figure 2A shows the peaks were individually separated at their respective retention times. The peak purity was monitored closely from the characteristic UV spectra of the four target compounds. The compounds showed maximum wavelengths (λ_max_) at around 270 and 340 nm, specifically isoorientin (270 and 350 nm), orientin (268 and 350 nm), vitexin (270 and 332 nm), and isovitexin (270 and 336 nm) [26]. Thus, UV/PDA is a valuable compound detector.

Figure 2B reveals the complexity of OPL crude extracts in the chromatogram. However, the hydrolysis helped to highlight the peaks of interest. The peaks of isoorientin, orientin, vitexin, and isovitexin were detected in both OPL extracts at the same retention times, as shown in Figure 2A. In addition, the characteristic fragmentation patterns of isoorientin, orientin, vitexin, and isovitexin in OPL extracts were confirmed with LC-MS/MS analysis (Figure S2) [27]. This suggests that no contaminants in the OPL extracts were eluted at the same retention time within the selected wavelength [11]. Therefore, this method was considered selective for quantitative analysis.

#### 3.1.2. Linearity, Limits of Detection (LOD), and Limits of Quantification (LOQ)

Linearity was validated based on the values of the correlation coefficients of isoorientin, orientin, vitexin, and isovitexin calibration curves. All the calibration curves showed a linear response with r^2^ > 0.999. Table 1 lists the concentration range, regression equation, and correlation coefficient obtained from the respective calibration curves together with the LOD and LOQ values. The LOD for isoorientin, orientin, vitexin, and isovitexin were 17.99, 30.22, 80.63, and 17.69 µg/mL, respectively, whereas the LOQ for the compounds were 54.52, 91.58, 244.35, and 54.61 µg/mL, respectively. The data suggest that the developed UHPLC method is sufficiently reliable for detecting and quantifying the target flavonoids present in OPL extracts.

#### 3.1.3. Accuracy, Precision, and Robustness

The accuracy of the developed method was assessed by recovery test, wherein known concentrations of flavonoid standards were added to the test by adding sample. The recoveries for isoorientin, orientin, vitexin, and isovitexin were 98.32–102.34%, 95.61–99.33%, 99.22–100.21%, and 100.68–102.79%, respectively. These recovery percentages were within the acceptable range (95–105%). Moreover, the RSD values of intra-day and inter-day precisions of the developed UHPLC method ranged from 0.04 to 1.74%, as shown in Table 2 Hence, the developed method was deemed precise as the obtained values were below 5%, which is the value that was suggested by ICH guidelines [26]. Furthermore, the robustness of the method was evaluated with the minor adjustment of UHPLC conditions such as column temperature, wavelength detector, and stability across different days. The *t*-test results revealed that the developed method was robust as there were no significant changes when the conditions were adjusted (*p* > 0.05). Overall, the developed UHPLC method was selective, accurate, repeatable, and robust, indicating that the method was appropriate for quantifying the luteolin and apigenin derivatives in the OPL extracts.

### 3.2. Application of UHPLC-UV/PDA Method for Quantification of Luteolin and Apigenin Derivatives in Various OPL Extracts

The OPL extracts were shown to contain 12 major flavonoid *C*-glycosides comprising six luteolin and six apigenin derivatives. Out of the 12 compounds, four compounds (isoorientin, orientin, vitexin, and isovitexin) were absolutely quantified by comparing with commercial standards, while the rest of the compounds, identified as luteolin and apigenin derivatives, were relatively quantified (Figure S1 and Table S1). The luteolin derivatives were assigned as luteolin-6,8-di-*C*-hexose (Isomer 1) (**1**), luteolin-6,8-di-*C*-hexose (Isomer 2) (**3**), isoorientin (**5**), orientin (**6**), luteolin-6-*C*-hexose-8-*C*-deoxyhexose (Isomer 1) (**7**), and luteolin-6-*C*-hexose-8-*C*-deoxyhexose (Isomer 2) (**9**). Meanwhile, the apigenin derivatives were assigned as apigenin-6,8-di-*C*-hexose (**2**), apigenin-6-*C*-pentose-8-*C*-hexose (Isomer 1) (**4**), apigenin-6-*C*-pentose-8-*C*-hexose (Isomer 2) (**8**), vitexin (**10**), isovitexin (**11**), and apigenin-6-*C*-hexose-8-*C*-deoxyhexose (**12**). The amounts of the luteolin and apigenin derivatives in the various OPL extracts were tabulated in Table 3 and Table 4, respectively. The total luteolin content (TLC) and total apigenin content (TAC) refer to the cumulative amount of luteolin and apigenin derivatives present in OPL extracts, respectively. However, hexane apparently could not extract any of the flavonoid-*C*-glycosides, whereas ethyl acetate could only extract luteolin-6,8-di-*C*-hexose (Isomer 1) (**1**), isoorientin (**5**), orientin (**6**), isovitexin (**11**), and apigenin-6-*C*-hexose-8-*C*-deoxyhexose (**12**) present in the OPL.

The present study revealed that the freeze-dried extracts possessed the highest amount of luteolin derivatives, with TLC values of 2.04–56.30 µg/mg, followed by oven-dried and shade-dried extracts with values of 2.15–51.87 and 2.10–43.26 µg/mg, respectively. Meanwhile, aqueous methanolic extracts contained the highest content of luteolin derivatives, with TLC values of 43.26–56.30 µg/mg, followed by absolute methanolic mixtures of methanol–ethyl acetate and ethyl acetate extracts with values of 30.44–42.14, 21.74–29.05, and 2.04–2.15 µg/mg, respectively. Among the identified luteolin derivatives in freeze-dried aqueous methanolic extracts, isoorientin (**5**) was found to be the highest, with a TLC value of 23.54 µg/mg, followed by orientin (**6**), luteolin-6-*C*-hexose-8-*C*-deoxyhexose (Isomer 1) (**7**), luteolin-6,8-di-*C*-hexose (Isomer 1) (**1**), luteolin-6-*C*-hexose-8-*C*-deoxyhexose (Isomer 2) (**9**), and luteolin-6,8-di-*C*-hexose (Isomer 2) (**3**), with values of 13.9, 9.06, 4.40, 3.70, and 1.70 µg/mg, respectively. Furthermore, aqueous methanol was able to extract the highest amount of apigenin derivatives, with TAC values ranging from 148.57–160.38 µg/mg, followed by absolute methanol, 1:1 ethyl acetate–methanol, and ethyl acetate with TAC values of 109.38–118.99, 62.08–99.14, and 1.84–2.14 µg VE/mg, respectively. For the freeze-dried aqueous methanolic extracts, apigenin-6-*C*-hexose-8-*C*-deoxyhexose (**12**) was found to be highest, with a value of 57.53 µg/mg, followed by apigenin-6,8-di-*C*-hexose (**2**), vitexin (**10**), apigenin-6-*C*-pentose-8-*C*-hexose (Isomer 1) (**4**), apigenin-6-*C*-pentose-8-*C*-hexose (Isomer 2) (**8**), and isovitexin (**11**), with values of 33.34, 32.62, 30.90, 4.04, and 1.95 µg/mg, respectively.

Overall, consistent with previous studies [6,28,29,30,31], quantitative analysis revealed that freeze-drying showed relatively better ability in preserving luteolin and apigenin derivatives in OPL. However, in most cases, oven-drying and shade-drying also showed comparable performance in preserving apigenin and luteolin derivatives, including isoorientin, orientin, vitexin, and isovitexin. The findings disclosed that luteolin and apigenin derivatives were preserved in all three drying methods, suggesting that the drying conditions applied in each of the drying methods were not deleterious to these compounds. Moreover, the trends revealed that as the polarity of the solvent increased, higher amounts of luteolin and apigenin derivatives could be extracted, indicating that these compounds possess moderate to high polarity. The solubility of the compounds of interest can be estimated using Hansen solubility parameters by understanding the basic structure that helps to estimate the polarity range of the solvent to use for extraction. Knowing the existence of several hydroxyl groups and sugar moieties attached to the aglycone of the flavone structure (Figure 1) [4,32], the addition of a water component to an organic polar solvent such as methanol helped to modify the polarity of the solvent mixture. This could explain the excellent performance of aqueous methanol in extracting luteolin and apigenin derivatives from the OPL matrix. These findings were consistent with previous studies, which also reported that modification of the conventional organic solvent system with aqueous elements enhanced the solvent polarity, enabling the extraction of polar compounds [24,33,34].

### 3.3. Polyphenolic Content and Antioxidant Activity in Various OPL Extracts

The TPC, TFC, DPPH, and NO free radical-scavenging activity of the OPL extracts produced using different combination of drying methods and extraction solvents are presented in Table 5. The TPC of OPL extracts ranged from 119.35 to 552.80 mg GAE/g, whereas the TFC values of OPL extracts ranged from 9.07 to 171.07 mg QCE/g. Both polyphenolic contents indicated freeze-drying as the most effective drying method to preserve the TPC and TFC in OPL compared to oven-drying and shade-drying, whereas aqueous methanol produced OPL extracts with highest amounts of TPC and TFC. Furthermore, the data revealed the significant effects of DPPH and NO inhibitions on the antioxidant activity of the respective OPL extracts. With respect to drying methods, freeze-dried extracts exhibited the most potent antioxidant activity with the lowest IC_50_ values for both DPPH and NO free radical-scavenging activity (14.02 and 14.29 µg/mL). Meanwhile, for different extraction solvents, methanol-containing solvent systems were able to produce OPL extracts with more potent antioxidant activity compared to hexane and ethyl acetate. Among the three methanolic extracts, the aqueous methanol extracts were the most potent extracts. These results indicate that freeze-drying and aqueous methanol were the most effective drying method and extraction solvent to preserve and extract antioxidant compounds such as phenolics and flavonoids from OPL, which were consistent with previous reports on other plant materials [22,24].

### 3.4. Partial Least Square Analysis (PLS) Correlation

To analyze the correlation between the relative quantities of the flavonoid and antioxidant activity of the samples, a PLS model was fitted to a unit variance-scaled dataset (dimension = 45 × 16, where X variables = 12 (relative quantity of 12 flavonoids) and Y variables = 4 (TPC and TFC values and 1/IC_50_ values for DPPH and NO radical-scavenging assays)). A two-component PLS model was obtained, with R^2^X (cumulative up to component 2), R^2^Y (cumulative up to component 2), and *Q^2^* (cumulative up to component 2) of 98.0%, 85.5%, and 83.8%, respectively. The model was cross-validated following the seven-fold cross-validation procedure. The cross-validation plots of the model with 200 times permutation tests (Figure S3) indicated that the model did not overfit the data.

**Figure 3:**
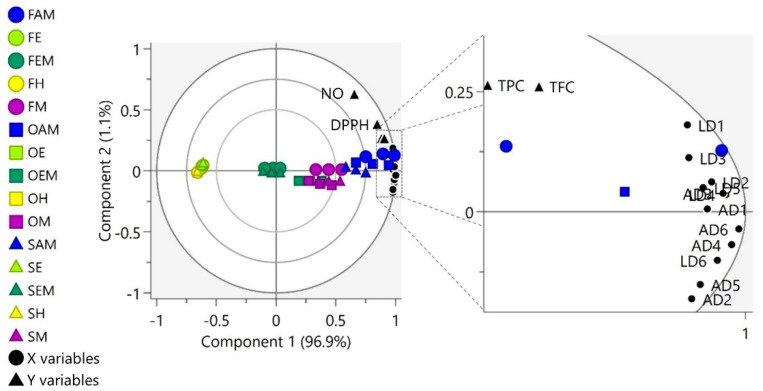
Biplot of the partial least square (PLS) model exhibiting correlation between relatively quantified luteolin and apigenin derivatives and antioxidant activity of OPL extracts obtained from various drying methods and extraction solvent systems. The ellipses (innermost to outermost) represent correlation-scaled loadings of values 0.50, 0.75, and 1.00, respectively. Abbreviations: F, freeze-dried; O, oven-dried; S, shade-dried; H, hexane; E, ethyl acetate; EM, ethyl acetate–methanol; M, absolute methanol; AM, aqueous methanol; TPC, total phenolic content; TFC, total flavonoid content; DPPH, 2,2-diphenyl-1-picrylhydrazyl; NO, nitric oxide; LD, luteolin derivatives; AD, apigenin derivatives; LD1, luteolin-6,8-di-*C*-hexose (Isomer 1); LD2, luteolin-6,8-di-*C*-hexose (Isomer 2); LD3, isoorientin; LD4, orientin; LD5, luteolin-6-*C*-hexose-8-*C*-deoxyhexose (Isomer 1); LD6, luteolin-6-*C*-hexose-8-*C*-deoxyhexose (Isomer 2); AD1, apigenin-6,8-di-*C*-hexose; AD2, apigenin-6-*C*-pentose-8-*C*-hexose (Isomer 1); AD3, apigenin-6-*C*-pentose-8-*C*-hexose (Isomer 2); AD4, vitexin; AD5, isovitexin; AD6, apigenin-6-*C*-hexose-8-*C*-deoxyhexose.

As depicted in Figure 3, the first component (explained variation = 96.9%) of the PLS model showed that the clustering of the samples was mainly influenced by the polarity of the extraction solvents, where samples extracted using highly polar solvents (methanol and aqueous methanol) were located at the positive side of the plot and samples extracted using non-polar solvent (hexane) were positioned at the negative side of the plot. On the other hand, samples extracted using moderately polar solvents (ethyl acetate and a combination of ethyl acetate and methanol) have average properties, as they were located near the origin of the plot. The second component (explained by variation = 1.1%) of the model further demarcated freeze-dried samples from most of the shade- and oven-dried samples, where 80% of the freeze-dried samples were located at the positive side of component 2 (with the exception of freeze-dried samples extracted using hexane).

All X and Y variables were projected at the positive side of the first component, specifically between the 0.75 and 1.00 correlation-scaled loading ellipses and close to the freeze- and oven-dried samples extracted using aqueous methanol (FAM and OAM). These findings revealed that FAM and OAM are the best combination of drying and extraction method to extract luteolin and apigenin derivatives, which are the flavonoids that have positive correlations with radical-scavenging antioxidant activity. These findings were in agreement with previous data that reported that flavonoid *C*-glycosides from various plant samples possess high antioxidant activity [4,22,24,25].

## 4. Conclusions

In this investigation, the validated UHPLC-UV/PDA method demonstrated that it is suitable and reliable for quantitative analysis of luteolin and apigenin derivatives, particularly orientin, isoorientin, vitexin, and isovitexin, present in various OPL extracts. The method offers good specificity, linearity, sensitivity, accuracy, precision, and robustness where the values obtained were within acceptable limits. Additionally, standardized OPL extract prepared by freeze-drying and extracted with aqueous methanol manifested greater ability to preserve and recover luteolin and apigenin derivatives with excellent antioxidant activity. Therefore, the study suggests the validated UHPLC-UV/PDA method could be applied in OPL-based nutraceutical and pharmaceutical industries by adopting orientin, isoorientin, vitexin, and isovitexin as chemical markers for quality control purposes.

## Figures and Tables

**Table 1:** Linearity, LOD, and LOQ of isoorientin, orientin, vitexin, and isovitexin.

Flavonoid	Concentration Range (µg/mL)	Regression Equation	Correlation Coefficient (R^2^)	LOD (µg/mL)	LOQ (µg/mL)
Isoorientin	16–500	y = 1931.7x + 3571.4	0.9999	17.99	54.52
Orientin	31–800	y = 2706.2x − 19677	0.9999	30.22	91.58
Vitexin	47–1500	y = 534.05x − 6500.5	0.9997	80.63	244.35
Isovitexin	16–500	y = 11136x + 13498	0.9999	17.69	53.61

**Table 2:** Recovery (%), relative standard deviation (RSD %) and *t*-test (*p*-value) of isoorientin, orientin, vitexin, and isovitexin.

Flavonoids	Concentration (µg/mL)	Recovery (%)	Precision (RSD %)		Robustness *p*-Value (*t*-Test)		
			Intra-Day	Inter-Day	CT (°C)	λ (nm)	Day
Isoorientin	High	102.34	1.25	0.01	0.06	0.07	0.37
	Medium	100.21	0.81	0.04			
	Low	98.32	1.49	0.66			
Orientin	High	95.61	0.45	0.32	0.15	0.07	0.24
	Medium	97.71	0.87	0.50			
	Low	99.33	0.34	0.28			
Vitexin	High	99.55	0.48	0.72	0.24	0.24	0.10
	Medium	99.22	0.05	0.56			
	Low	100.21	1.29	1.74			
Isovitexin	High	102.79	1.15	1.31	0.15	0.18	0.14
	Medium	101.18	0.09	1.49			
	Low	100.68	0.17	0.80			

*p* > 0.05 = not significant; CT—column temperature, λ—wavelength detector.

**Table 3:** Relative quantification of luteolin derivatives in OPL extracts obtained from various drying methods and solvent systems.

Drying	Solvent	Peak 1	Peak 3	* Peak 5	* Peak 6	Peak 7	Peak 9	TLC (µg/mg)
O	E	0.61 ± 0.11 ^Aa^	ND	0.74 ± 0.09 ^Aa^	0.80 ± 0.23 ^Aa^	ND	ND	2.15 ± 0.43 ^Aa^
	EM	2.22 ± 0.18 ^Ab^	0.97 ± 0.19 ^Aa^	10.14 ± 0.85 ^Ab^	7.99 ± 0.23 ^Ab^	5.35 ± 0.79 ^Aa^	2.38 ± 0.31 ^Aa^	29.05 ± 2.55 ^Ab^
	M	2.36 ± 0.14 ^Ab^	0.98 ± 0.10 ^Aa^	10.05 ± 1.17 ^Ab^	8.16 ± 0.41 ^Ab^	5.42 ± 0.42 ^Aa^	3.47 ± 0.50 ^Ab^	30.44 ± 2.74 ^Ab^
	AM	3.90 ± 0.15 ^Ac^	1.66 ± 0.12 ^Ab^	21.85 ± 2.19 ^Ac^	13.26 ± 1.18 ^Ac^	7.27 ± 1.04 ^Ab^	3.93 ± 0.39 ^Ab^	51.87 ± 5.07 ^Ac^
F	E	0.60 ± 0.13 ^Aa^	ND	0.72 ± 0.10 ^Aa^	0.72 ± 0.20 ^Aa^	ND	ND	2.04 ± 0.43 ^Aa^
	EM	1.47 ± 0.16 ^Bb^	0.89 ± 0.13 ^Aa^	9.66 ± 1.13 ^Ab^	6.51 ± 0.10 ^Bb^	3.20 ± 0.22 ^Ba^	1.63 ± 0.09 ^Ba^	23.36 ± 1.83 ^Bb^
	M	2.49 ± 0.21 ^Ac^	1.20 ± 0.08 ^Ab^	18.58 ± 1.19 ^Bc^	11.48 ± 0.89 ^Bc^	5.64 ± 0.69 ^Ab^	2.75 ± 0.48 ^Ab^	42.14 ± 3.54 ^Bc^
	AM	4.40 ± 0.18 ^Bd^	1.70 ± 0.05 ^Ac^	23.54 ± 3.39 ^Ad^	13.90 ± 1.30 ^Ad^	9.06 ± 2.11 ^Bc^	3.70 ± 0.29 ^Ac^	56.30 ± 7.32 ^Ad^
S	E	0.65 ± 0.15 ^Aa^	ND	0.70 ± 0.05 ^Aa^	0.75 ± 0.15 ^Aa^	ND	ND	2.10 ± 0.35 ^Aa^
	EM	1.32 ± 0.11 ^Bb^	0.75 ± 0.09 ^Ba^	8.36 ± 1.15 ^Ab^	6.38 ± 0.21 ^Bb^	3.14 ± 0.39 ^Ba^	1.79 ± 0.24 ^Ba^	21.74 ± 2.19 ^Bb^
	M	2.32 ± 0.14 ^Ac^	1.09 ± 0.12 ^Ab^	15.54 ± 2.19 ^Bc^	10.63 ± 1.08 ^Bc^	4.92 ± 0.81 ^Ab^	2.96 ± 0.19 ^Ab^	37.46 ± 4.53 ^Bc^
	AM	3.25 ± 0.13 ^Cd^	1.24 ± 0.07 ^Bb^	16.39 ± 1.59 ^Bc^	11.20 ± 1.12 ^Ac^	7.52 ± 1.19 ^Ac^	3.66 ± 0.26 ^Ac^	43.26 ± 4.36 ^Bc^

Values marked with different upper-case letters (A,B,C) indicate comparison between drying methods for the same solvent. Values marked with different lower-case letters (a,b,c,d) indicate comparison between solvents for the same drying method. The significant difference is set at *p* < 0.05. Abbreviations: F, freeze-dried; O, oven-dried; S, shade-dried; H, hexane; E, ethyl acetate; EM, ethyl acetate–methanol; M, absolute methanol; AM, aqueous methanol; TLC, total luteolin content; Peak 1, luteolin-6,8-di-*C*-hexose (Isomer 1); Peak 3, luteolin-6,8-di-*C*-hexose (Isomer 2); Peak 5, isoorientin; Peak 6, orientin; Peak 7, luteolin-6-*C*-hexose-8-*C*-deoxyhexose (Isomer 1); Peak 9, luteolin-6-*C*-hexose-8-*C*-deoxyhexose (Isomer 2). ND—not detected. * indicates absolute quantification using respective standard. Not all hexanoic extracts could extract all quantified luteolin and apigenin derivatives.

**Table 4:** Relative quantification of apigenin derivatives in OPL extracts obtained from various drying methods and solvent systems.

Drying	Solvent	Peak 2	Peak 4	Peak 8	* Peak 10	* Peak 11	Peak 12	TAC (µg /mg)
O	E	ND	ND	ND	ND	0.01 ± 0.02 ^Aa^	1.83 ± 0.13 ^Aa^	1.84 ± 0.15 ^Aa^
	EM	17.50 ± 1.31 ^Aa^	3.80 ± 0.45 ^Aa^	17.65 ± 3.01 ^Aa^	22.04 ± 1.11 ^Aa^	1.54 ± 0.11 ^Ab^	36.61 ± 2.34 ^Ab^	99.14 ± 8.33 ^Ab^
	M	22.46 ± 0.85 ^Ab^	3.61 ± 0.31 ^Aa^	17.95 ± 3.20 ^Aa^	25.50 ± 2.32 ^Ab^	1.62 ± 0.19 ^Ab^	40.70 ± 3.18 ^Ab^	111.84 ± 10.1 ^Ab^
	AM	30.93 ± 2.11 ^Ac^	4.77 ± 0.41 ^Ab^	29.11 ± 5.12 ^Ab^	29.33 ± 2.18 ^Ac^	1.66 ± 0.25 ^Ab^	57.99 ± 4.12 ^Ac^	153.79 ± 14.2 ^Ac^
F	E	ND	ND	ND	ND	0.02 ± 0.01 ^Aa^	1.83 ± 0.11 ^Aa^	1.85 ± 0.12 ^Aa^
	EM	10.78 ± 1.21 ^Ba^	1.95 ± 0.21 ^Ba^	12.76 ± 2.21 ^Ba^	14.07 ± 1.13 ^Ba^	0.65 ± 0.14 ^Bb^	21.87 ± 2.19 ^Bb^	62.08 ± 7.09 ^Bb^
	M	18.95 ± 2.10 ^Ab^	3.39 ± 0.12 ^Ab^	23.66 ± 4.76 ^Bb^	21.62 ± 2.10 ^Ab^	1.49 ± 0.15 ^Ac^	40.27 ± 3.91 ^Ac^	109.38 ± 13.1 ^Ac^
	AM	33.34 ± 2.31 ^Ac^	4.04 ± 0.25 ^Ac^	30.90 ± 3.14 ^Ac^	32.62 ± 1.98 ^Ac^	1.95 ± 0.11 ^Bd^	57.53 ± 4.32 ^Ad^	160.38 ± 12.1 ^Ad^
S	E	ND	ND	ND	ND	0.001 ± 0.02 ^Ba^	2.14 ± 0.20 ^Aa^	2.14 ± 0.22 ^Aa^
	EM	12.55 ± 0.75 ^Ba^	2.24 ± 0.18 ^Ba^	10.89 ± 2.25 ^Ba^	13.41 ± 1.41 ^Ba^	0.67 ± 0.13 ^Bb^	25.68 ± 2.11 ^Bb^	65.44 ± 6.83 ^Bb^
	M	23.09 ± 1.11 ^Ab^	4.22 ± 0.20 ^Bb^	20.24 ± 5.10 ^Bb^	23.33 ± 2.15 ^Ab^	1.69 ± 0.10 ^Ac^	46.42 ± 3.91 ^Ac^	118.99 ± 12.5 ^Ac^
	AM	35.16 ± 1.95 ^Ac^	3.72 ± 0.31 ^Ac^	24.11 ± 4.41 ^Bb^	28.97 ± 2.41 ^Ac^	1.92 ± 0.12 ^Bd^	54.69 ± 4.81 ^Ac^	148.57 ± 14.0 ^Ad^

Values marked with different upper-case letters (A,B) indicate comparison between drying methods for the same solvent. Values marked with different lower-case letters (a,b,c,d) indicate comparison between solvents for the same drying method. The significant difference is set at *p* < 0.05. Abbreviations: F, freeze-dried; O, oven-dried; S, shade-dried; H, hexane; E, ethyl acetate; EM, ethyl acetate–methanol; M, absolute methanol; AM, aqueous methanol;TAC, total apigenin content; Peak 2, apigenin-6,8-di-*C*-hexose; Peak 4, apigenin-6-*C*-pentose-8-*C*-hexose (Isomer 1); Peak 8, apigenin-6-*C*-pentose-8-*C*-hexose (Isomer 2); Peak 10, vitexin; Peak 11, isovitexin; Peak 12, apigenin-6-*C*-hexose-8-*C*-deoxyhexose. ND—not detected. * indicates absolute quantification using respective standard. Not all hexanoic extracts could extract all quantified luteolin and apigenin derivatives.

**Table 5:** Influence of drying methods and solvent systems on polyphenolic content and antioxidant activity of OPL.

Drying	Solvent	TPC (mg GAE/g)	TFC (mg QCE/g)	DPPH (IC_50_ µg/mL)	NO (IC_50_ µg/mL)
O	H	119.35 ± 2.86 ^Aa^	10.46 ± 2.13 ^Aa^	299.42 ± 5.57 ^Aa^	72.30 ± 2.49 ^Aa^
	E	226.13 ± 5.77 ^Ab^	13.32 ± 2.05 ^Aa^	264.08 ± 8.56 ^Ab^	43.95 ± 5.40 ^Ab^
	EM	337.98 ± 2.47 ^Ac^	57.26 ± 2.19 ^Ab^	45.97 ± 0.36 ^Ac^	48.88 ± 4.44 ^Ab^
	M	381.73 ± 4.75 ^Ad^	57.58 ± 3.28 ^Ab^	42.63 ± 0.22 ^Ac^	49.63 ± 4.87 ^Ab^
	AM	393.27 ± 1.65 ^Ad^	107.66 ± 2.36 ^Ac^	19.32 ± 2.69 ^Ad^	19.34 ± 3.33 ^Ac^
F	H	124.88 ± 4.95 ^Aa^	9.93 ± 0.10 ^Aa^	762.85 ± 9.63 ^Aa^	67.45 ± 3.67 ^Aa^
	E	237.44 ± 3.50 ^Ab^	11.28 ± 0.01 ^Bb^	259.76 ± 4.67 ^Ab^	31.37 ± 2.03 ^Bb^
	EM	310.77 ± 3.40 ^Ac^	57.74 ± 0.63 ^Ac^	21.86 ± 0.28 ^Bc^	43.33 ± 6.23 ^Ac^
	M	460.83 ± 7.55 ^Bd^	91.23 ± 1.44 ^Bd^	35.24 ± 1.32 ^Bd^	41.20 ± 3.71 ^Ac^
	AM	552.80 ± 9.53 ^Be^	171.07 ± 0.63 ^Be^	14.29 ± 0.29 ^Bc^	14.02 ± 1.65 ^Bd^
S	H	139.23 ± 6.54 ^Aa^	9.07 ± 0.16 ^Ba^	917.34 ± 7.33 ^Ba^	70.84 ± 2.78 ^Aa^
	E	198.33 ± 8.88 ^Ab^	13.31 ± 0.06 ^Ab^	411.24 ± 7.43 ^Bb^	58.16 ± 4.90 ^Cb^
	EM	260.17 ± 1.88 ^Bc^	87.98 ± 1.04 ^Bc^	55.86 ± 0.79 ^Cc^	65.32 ± 7.96 ^Bb^
	M	251.55 ± 4.74 ^Cc^	82.50 ± 0.63 ^Cd^	44.56 ± 0.65 ^Ac^	49.63 ± 2.21 ^Ac^
	AM	404.11 ± 1.10 ^Ad^	83.93 ± 0.41 ^Cd^	30.44 ± 0.61 ^Cd^	20.15 ± 0.92 ^Ad^

Values marked with different upper-case letters (A,B,C) indicate comparison between drying methods for the same solvent. Values marked with different lower-case letters (a,b,c,d,e) indicate comparison between solvents for the same drying method. DPPH IC_50_ of quercetin and gallic acid were 6.21 and 2.09 µg/mL, respectively, whereas NO IC_50_ of quercetin and gallic acid were 10.30 and 12.03 µg/mL, respectively. Abbreviations: F, freeze-dried; O, oven-dried; S, shade-dried; H, hexane; E, ethyl acetate; EM, ethyl acetate–methanol; M, absolute methanol; AM, aqueous methanol; TPC, total phenolic content; TFC, total flavonoid content; DPPH, 2,2-diphenyl-1-picrylhydrazyl; NO, nitric oxide free radical-scavenging activity.

## Supplementary Materials

The following are available online, Figure S1: Peak assignment for luteolin and apigenin derivatives in UV chromatograms (340 nm) of OPL extracts (various combination of drying methods and extraction solvent systems), Figure S2: LC-MS/MS spectra (ESI, negative mode) of the various OPL extracts, Table S1: Identification of phytoconstituents in aqueous methanolic OPL extracts (different drying methods and solvent systems) by UHPLC-MS/MS and UHPLC-UV/PDA methods, Figure S3: Cross-validation plots of PLS model with 200 times permutation tests. Plot for Y-variable TPC (A), TFC (**B**), 1/IC_50_ DPPH (**C**), and 1/IC_50_ NO (**D**).
