# Supplementary material for: UHPLC-UV/PDA Method Validation for Simultaneous Quantification of Luteolin and Apigenin Derivatives from Elaeis guineensis Leaf Extracts: An Application for Antioxidant Herbal Preparation
Source: Molecules. 2021 Feb 19;26(4):1084. doi: 10.3390/molecules26041084 (PMC7922162; doi:10.3390/molecules26041084)
Supplement: Supplementary file 1 [file molecules-26-01084-s001.pdf]

molecules

MDPI

Supplementary Materials

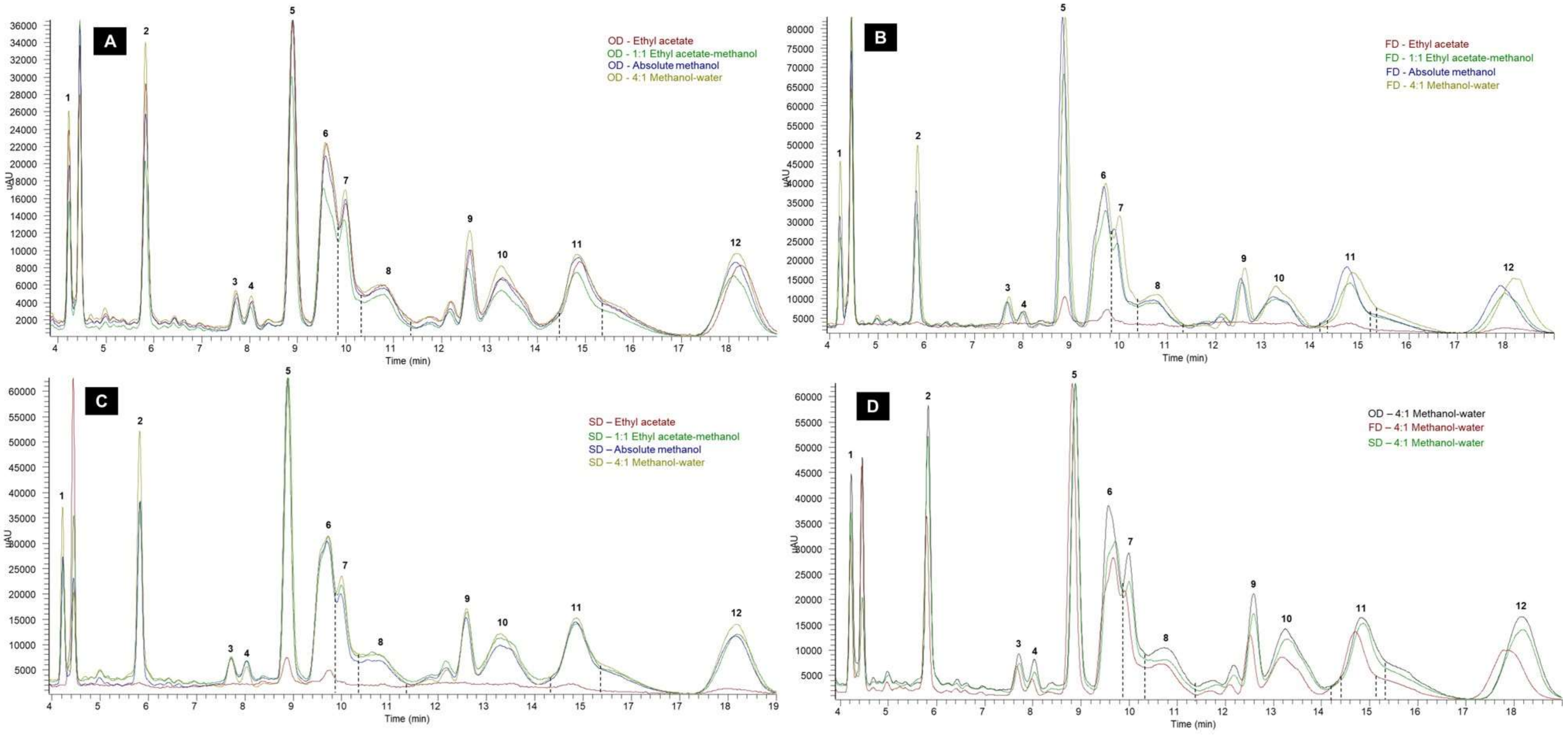

Figure S1 Peak assignment for luteolin and apigenin derivatives in OPL extracts after subjected to different drying methods and extraction solvents in UV chromatograms at 340 nm. (A) Oven dry – solvents, (B) Freeze dry – solvents, (C) Shade dry – solvents and (D) Drying methods – 4:1 Methanol-water. Identified flavonoid *C*-glycosides: **1**, luteolin-6,8-di-*C*-hexose (Isomer 1); **2**, apigenin-6,8-di-*C*-hexose; **3**, luteolin-6,8-di-*C*-hexose (Isomer 2); **4**, apigenin-6-*C*-pentose-8-*C*-hexose (Isomer 1); **5**, isoorientin; **6**, orientin; **7**, luteolin-6-*C*-hexose- 8-*C*-deoxyhexose (Isomer 1); **8**, apigenin-6-*C*-pentose-8-*C*-hexose (Isomer 2); **9**, luteolin-6-*C*-hexose- 8-*C*-deoxyhexose (Isomer 2); **10**, vitexin; **11**, isovitexin; **12**, apigenin-6-*C*-hexose-8-*C*-deoxyhexose.

Table S1 Identification of phytoconstituents in aqueous methanolic OPL extracts at different drying methods and solvents by UHPLC-MS/MS and UHPLC-UV/PDA method

**Peak**	**t_R (min)**	**λ_{max}, (nm)**	**$[M\text{-}H]^-$ (m/z)**	**Formula**	**Key MS/MS fragments (m/z)**	**Compound**	**OD**					**FD**					**SD**				
							**1**	**2**	**3**	**4**	**5**	**1**	**2**	**3**	**4**	**5**	**1**	**2**	**3**	**4**	**5**
1	4.17	272, 348	609.1411	$C_{27}H_{30}O_{16}$	519.1104 489.0998, 429.0786, 399.0696, 369.0585	Luteolin-6,8-di-*C*-hexose	-	+	+	+	+	-	+	+	+	+	-	+	+	+	+
2	5.88	272, 336	593.1464	$C_{27}H_{30}O_{15}$	503.1155, 473.1051, 383.0739, 353.0638	Apigenin-6,8-di-*C*-hexose	-	-	+	+	+	-	-	+	+	+	-	-	+	+	+
3	7.72	272, 346	609.1411	$C_{27}H_{30}O_{16}$	489.1001, 429.0789, 399.0679, 369.0604	Luteolin-6,8-di-C-hexose	-	-	+	+	+	-	-	+	+	+	-	-	+	+	+
4	8.10	272, 334	563.1359	$C_{26}H_{28}O_{14}$	473.1053, 443.0949, 383.0742, 353.0639	Apigenin-6-C-pentose-8-C-hexose	-	-	+	+	+	-	-	+	+	+	-	-	+	+	+
5	8.86	270, 348	447.0896	$C_{21}H_{20}O_{11}$	357.0588, 339.0480, 327.0483, 297.0379, 285.0381	Isoorientin (Luteolin-6-C-hexose)	-	+	+	+	+	-	+	+	+	+	-	+	+	+	+
6	9.60	270, 350	447.0896	$C_{21}H_{20}O_{11}$	357.0587, 339.0476, 327.0485, 297.0378, 285.0380	Orientin (Luteolin-8-C-hexose)	-	+	+	+	+	-	+	+	+	+	-	+	+	+	+
7	10.00	270, 348	593.1464	$C_{27}H_{30}O_{15}$	473.1049, 429.0792, 369.0590, 357.0589, 327.0485	Luteolin-6-*C*-hexose- 8-*C*-deoxyhexose	-	-	+	+	+	-	-	+	+	+	-	-	+	+	+
8	10.60	274, 334	563.1359	$C_{26}H_{28}O_{14}$	503.1168, 473.1056, 443.0950, 383.0743, 353.0639	Apigenin-6-*C*-pentose-8-C-hexose	-	-	+	+	+	-	-	+	+	+	-	-	+	+	+
9	12.60	272, 336	593.1464	$C_{27}H_{30}O_{15}$	473.1067, 413.0846, 369.0590, 357.0589, 293.0434	Luteolin-6-*C*-hexose- 8-*C*-deoxyhexose	-	-	+	+	+	-	-	+	+	+	-	-	+	+	+
10	13.44	270, 338	431.0947	$C_{21}H_{20}O_{10}$	341.0639, 323.0529, 311.0536, 283.0589	Vitexin (Apigenin-6-C-hexose)	-	-	+	+	+	-	-	+	+	+	-	-	+	+	+
11	14.85	270, 338	431.0947	$C_{21}H_{20}O_{10}$	341.0638, 323.0536, 311.0536, 283.0588	Isovitexin (Apigenin-8-C-hexose)	-	+	+	+	+	-	+	+	+	+	-	+	+	+	+
12	18.19	270, 338	577.1306	$C_{27}H_{30}O_{14}$	457.1098, 413.0845, 353.0630, 341.0640, 311.0536, 293.0432	Apigenin-6-*C*-hexose-8-*C*-deoxyhexose	-	+	+	+	+	-	+	+	+	+	-	+	+	+	+

1; hexane; 2, ethyl acetate; 3, 1:1 ethyl acetate-methanol; 4, absolute methanol; 5, 4:1 methanol-water; +, present; -, absent

molecules

MDPI

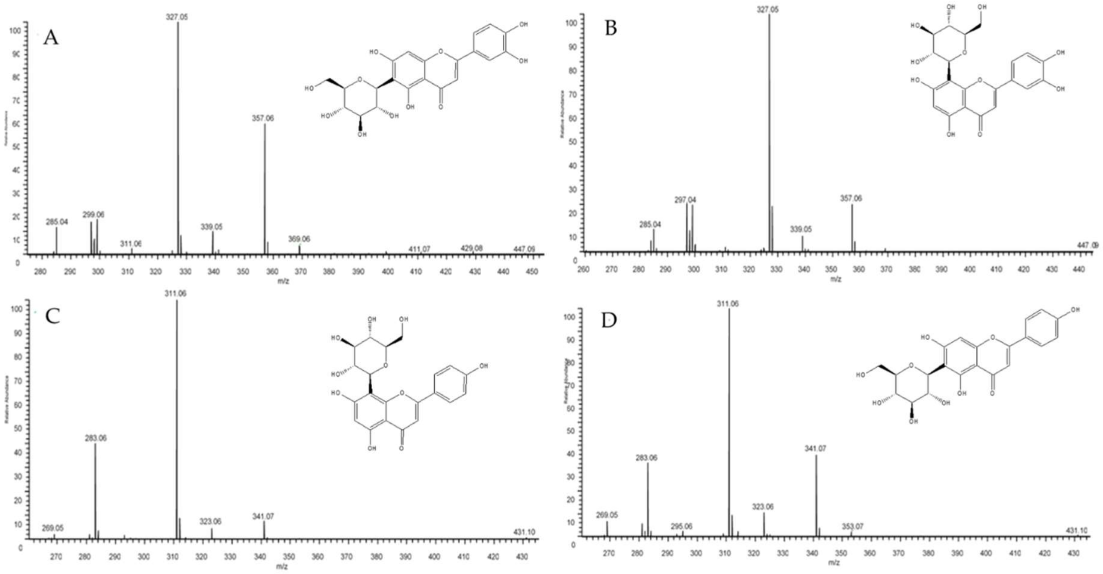

Figure S2 LC-MS/MS spectra (ESI, negative mode) of OPL extract which the peak identified as (A) isoorientin; (B) orientin; (C) vitexin and (D) isovitexin. Characteristic fragment ions [M-H-90]- (m/z 357.06) and [M-H-120]- (m/z 327.05) of isoorientin and orientin while [M-H-90]- (m/z 341.07) and [M-H-120]- (m/z 311.06) of vitexin and isovitexin

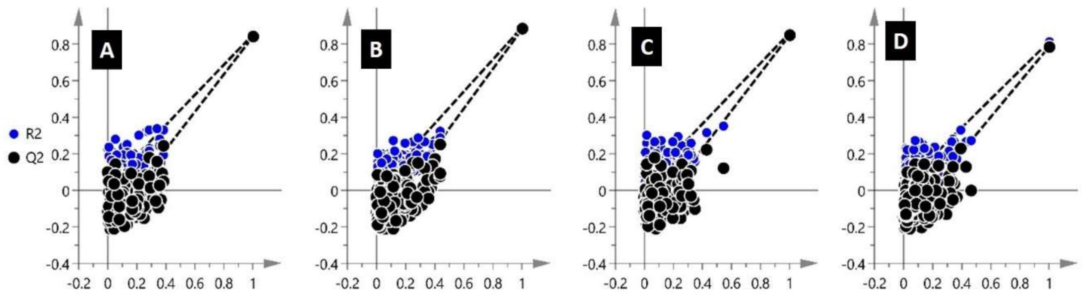

Figure S3 Cross-validation plots of PLS model with 200 times permutation tests. Plot for Y-variable TPC (A); TFC (B); $1/IC_{50}$ DPPH (C) and $1/IC_{50}$ NO (D).
